# Anisotropic Diffusion of Elongated Particles in Active Coherent Flows

**DOI:** 10.3390/mi15020199

**Published:** 2024-01-28

**Authors:** Dongdong Li, Yanan Liu, Hao Luo, Guangyin Jing

**Affiliations:** School of Physics, Northwest University, Xi’an 710127, China

**Keywords:** active flows, anisotropic particles, superdiffusion

## Abstract

The study of particle diffusion, a classical conundrum in scientific inquiry, holds manifold implications for various real-world applications. Particularly within the domain of active flows, where the motion of self-propelled particles instigates fluid movement, extensive research has been dedicated to unraveling the dynamics of passive spherical particles. This scrutiny has unearthed intriguing phenomena, such as superdiffusion at brief temporal scales and conventional diffusion at longer intervals. In contrast to the spherical counterparts, anisotropic particles, which manifest directional variations, are prevalent in nature. Although anisotropic behavior in passive fluids has been subject to exploration, enigmatic aspects persist in comprehending the interplay of anisotropic particles within active flows. This research delves into the intricacies of anisotropic passive particle diffusion, exposing a notable escalation in translational and rotational diffusion coefficients, as well as the superdiffusion index, contingent upon bacterial concentration. Through a detailed examination of particle coordinates, the directional preference of particle diffusion is not solely dependent on the particle length, but rather determined by the ratio of the particle length to the associated length scale of the background flow field. These revelations accentuate the paramount importance of unraveling the nuances of anisotropic particle diffusion within the context of active flows. Such insights not only contribute to the fundamental understanding of particle dynamics, but also have potential implications for a spectrum of applications.

## 1. Introduction

Active coherent flows represent a novel class of non-equilibrium systems observed in various active materials, such as bacteria and cells [1,2,3,4,5,6,7,8], flocks of birds and schools of fish, among other animals [9,10,11,12,13,14,15], artificially self-propelled colloidal particles [16,17,18,19,20,21], or filamentous proteins [22,23]. Since the theoretical understanding of Brownian motion in a thermal bath by Einstein, the motion of tracer particles in such active flows has been a topic of significant interest [24,25]. These reports not only contribute to unveiling the non-equilibrium nature of active coherent flows, but also provide a crucial understanding towards the efficient mixing, transport, and intercellular communication of biological substances [26,27,28,29,30,31,32,33,34]. For example, by analyzing the motion and diffusion of fluorescent bacteria in active flows, it is revealed that individual bacteria exhibit superdiffusive motion within the population. Bacterial cluster formation shifts their motion from Brownian to a Lévy walk pattern. The investigation of nanoscale particle dynamics in a bacterial fluid further confirms that both the fluid driven by bacteria and its constituents follow Lévy walk characteristics. This dual-layer Lévy model suggests the enhanced long-distance transport of substances and information among bacterial clusters [35]. In response to environmental stressors such as antibiotics, bacteria have evolved adaptive strategies to survive. It has been observed that, as an adaptation strategy to cope with deteriorating survival conditions induced by factors like antibiotics, bacteria tend to elongate their body length. Interestingly, within the bacterial population, a minute proportion of elongated bacterial members can significantly enhance collective flow within the group [36]. This discovery provides novel avenues for investigating the regulation of collective behaviors in bacterial communities and the mechanisms underlying microbial antibiotic resistance. The diffusion of spherical passive particles in these active coherent flows has been extensively studied, revealing superdiffusion in the short term and normal diffusion over longer durations [2,27,37,38,39,40,41,42,43,44,45,46].

However, in biological systems, in addition to the spherical symmetric structure, particles commonly exhibit shapes that are anisotropic, such as ellipsoids, fibers [47], and contractile proteins [48]. This anisotropic nature results in the dynamic behaviors of particles that are markedly distinct from those of spherical particles, including directional variations and intricate coupling between translational and rotational motion. The behavior of anisotropic particles in passive fluids has been extensively studied theoretically and experimentally [49,50,51,52,53,54,55]. One year after the development of spherical particle theory, Albert Einstein extended Brownian dynamics to include rotational freedom, leading to the exploration of ellipsoidal particle diffusion. Francis Perrin quantified these observations in 1930 [56,57]. Vasanthi et al. used molecular dynamics simulations to study ellipsoidal particles within spherical ones, finding a linear relationship between translational diffusion coefficients and the particle aspect ratio. Vasanthi suggested that, initially, particles exhibit anisotropic diffusion dominated by translation. As time progresses, rotation becomes influential, resulting in a gradual transition from anisotropic to isotropic diffusion at long timescales [58,59]. Han et al. observed the quasi-two-dimensional Brownian motion of ellipsoidal particles in water, confirming a transition from short-term anisotropic to long-term isotropic diffusion in the laboratory coordinate system. However, in the intrinsic coordinate system, particle diffusion remains anisotropic, without a transition to isotropy [60,61].

Recently, there have been experimental studies focusing on the dynamics of anisotropic passive particles in active flows [62,63,64,65,66,67]. In comparison to Brownian motion scenarios, it has been observed that, in active flows, particles exhibit anomalous coupled diffusion behavior, specifically diffusing preferentially along their minor axis. Moreover, this anomalous coupling behavior intensifies with an increase in the number density of active particles in the background flow field. Through investigations into various active flows, it is proposed that this anomalous coupling arises due to the unique structure of the flow field. However, certain mysteries persist regarding the diffusion of anisotropic particles in active flow, such as whether the coupling between the translational and rotational motion of tracer particles is influenced by particle size and the flow structure of the background flow field, which has yet to be systematically explored in experiments.

Here, we employed non-motile bacteria of *Escherichia coli* (*E. coli*, antibiotic-cultured strain AW405) to obtain individuals with rod-like bodies of varying lengths. The flagella of the passive bacteria were selectively removed by repetitive aspiration with a micropipette. Active coherent flows with varying characteristic lengths, the correlation length of the velocity field, were generated by employing another type of swimming bacteria, *Bacillus subtilis* (strain BS168) at different concentrations. Different lengths of passive rod-shaped bacteria were introduced into the active coherent flow, and the diffusion behavior of the rod-shaped bacteria in the active coherent flow was investigated within a quasi-two-dimensional cylindrical chamber. In the laboratory coordinate system, we focused on the translational diffusion coefficient, rotational diffusion coefficient, and superdiffusion exponent of the passive rod-shaped particles in the bacterial active flow, examining their relationships with the bacterial concentration and particle length. We observed that both translational diffusion coefficient $D_{T}$ and rotational diffusion coefficient $D_{R}$ monotonically increased with an increase in bacterial concentration. Additionally, at the same bacterial concentration, $D_{T}$ and $D_{R}$ both exhibited a monotonic decrease with an increase in particle length, while the superdiffusion exponent index showed no dependence on the length of the passive particles. Subsequently, in the body coordinate system, an analysis of the mean-squared displacement of the passive particles revealed that we found that not all particles exhibited anomalous coupled diffusion behavior. In other words, the particles did not uniformly prefer diffusion along the short axis. We associated the particle diffusion with the flow structure length coupling of the active coherent flow and determined its dependence on the body size of the non-motile bacteria.

## 2. Materials and Methods

### 2.1. Experimental Setup

**Figure 1:**
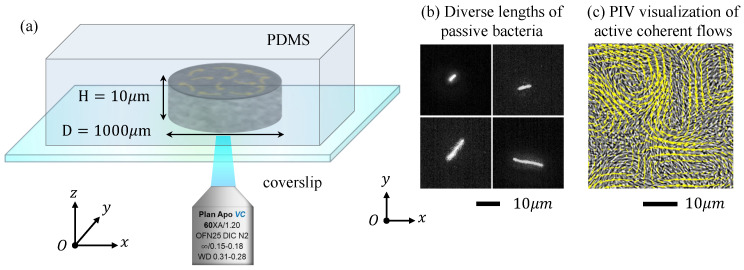
Experimental setup. (**a**) In a thin cylindrical chamber with a height of 10 μm and a diameter of 1000 μm, we investigated the dynamics of AW405 passive rod–shaped bacteria of varying lengths in the active flow of the BS168 suspension. (**b**) Different lengths of AW405 passive rod–shaped bacteria, with a constant width, were captured under fluorescent illumination. After selectively removing the flagella and staining, only the bacterial heads were visible, confirming the passive rod–like characteristics. (**c**) The bright–field snapshot of the bacterial suspension was recorded and analyzed by PIV measurements, thus obtaining the velocity field.

The entire experimental observation process took place within a thin cylindrical cavity on a Polydimethylsiloxane (PDMS) chamber, as illustrated in Figure 1. The cavity had a height of 10 μm and a diameter of 1000 μm. Concentrated bacteria (strain BS168) were used to generate active flows with coherent flow structures, and bacteria (strain AW405) with fluorescent dye were used as the non-motile elongated particles. The motility of AW405 was eliminated by removing the flagella, and their length can be tuned by adding an antibiotic. A drop of a mixed solution of concentrated BS168 and extremely dilute AW405 was introduced onto the glass cover slip and finally sealed with the PDMS chamber. The PDMS chamber was gas permeable, thus maintaining the motility of the bacteria for a certain time of around 30 min. We performed image acquisition with an inverted fluorescence microscope (Nikon Ti2-E, Nikon Corp, Tokyo, Japan) equipped with a high-speed camera (ORCA-Flash4.0 V3, Hamamatsu Photonics, Hamamatsu , Japan), focusing at the middle height of the channel. Two objective lenses of 40× (NA = 0.6) and 60× (NA = 1.2) were used with a visual field of about 328 μm×328 μm (0.16 μm/px) and 225 μm×225 μm (0.11 μm/px). Then, the movements of the fluorescent AW405 and non-fluorescent BS168 bacteria could be recorded in the fluorescent field and bright field, respectively, at a corresponding frame rate of 25 Hz and 50 Hz, with the same resolution of 2048 × 2044 px. The continuous sequence of bright-field images captured by the microscope provided the flow field information of the bacterial activity throughout the entire field. The PIVlabs macro in MATLAB 2020a analysis allowed us to obtain the velocity field from the raw video. Throughout all the PIV measurements, key parameters such as the window size and step size were set to 128 and 32 px, respectively. Subsequently, the optimal interrogation window size between the adjacent frames was calibrated to be 43.52 μm by 43.52 μm with a minimum lattice spacing of the velocity field 10.88 μm. The velocity field shown in Figure 1 illustrates the velocity vectors at each lattice. In addition, the spatial correlation of the bacterial activity flow structures could be determined using the velocity correlation function $C_{\nu \nu }\left(\delta r\right)=\langle v\left(r\right)\cdot v\left(r+\delta r\right)/v^{2}\left(r\right)\rangle$, where denotes the spatiotemporal averaging. By fitting the data to the exponential decay function $e^{-\delta r/\lambda }$, the correlation length λ could be calculated.

### 2.2. Bacterial Culture and Sample Preparation

**Bacterial strains and culture conditions:** Two bacterial strains, *E. coli* (strain AW405) and *Bacillus subtilis* (strain BS168), were employed in this study. Standard culture protocols were followed for both strains. Initially, approximately 10 μL of the bacterial suspension (aspirated from a cryotube stored at −20 °C) was cultivated overnight (approximately 12 h) with shaking (200 rpm) in 20 mL Luria-Bertani broth (containing 1.0% (*w*/*v*) tryptone, 1.0% (*w*/*v*) NaCl, and 0.5% (*w*/*v*) yeast) at 30 °C. Subsequently, 100 μL of the overnight culture was diluted 1:100 in Luria-Bertani broth and further incubated with shaking at 30 °C. After 2 h of incubation, cefotaxime (100 μg/mL) was added to the AW405 culture to inhibit cell division while allowing normal growth, leading to gradual elongation of the bacterial cells. Upon achieving the desired length, fluorescent labeling was performed. The BS168 culture was shaken for approximately 6 h until optimal bacterial motility was observed, resulting in a cell density of approximately $10^{8}$ cells/mL, as determined by optical densitometry at a wavelength of 600 nm. To maintain optimal bacterial motility and inhibit cell division, the bacterial suspension was washed twice with motility buffer (MB, 0.01 M potassium phosphate, 0.067 M NaCl, 10 M EDTA, pH = 7.0). After centrifugation to remove the supernatant, the pellet was resuspended in an appropriate volume of MB buffer. The optical density (OD) of the bacterial suspension was determined and recorded, with the volume fraction at the standard concentration $n_{0}\approx 8\times 10^{8}$ cells/mL being 0.1% and OD ∼1.

**Staining of AW405 cell bodies and flagella:** The staining procedure for AW405 cell bodies and flagella involved the following steps: First, the bacterial suspension was washed three times with motility buffer (MB). Subsequently, under subdued light conditions, the suspension was introduced to a mixture containing 5% sodium bicarbonate (1 M, pH = 7.8) and 2% Fluor^TM^ 488 (Thermo Fisher Alexa, Waltham, MA, USA) in dimethyl sulfoxide (DMSO) (99.9%, Sigma-Aldrich, Dorset, UK). The resulting mixture was gently shaken on a shaker (200 rpm, 30 °C) for 30 min. After shaking, the bacterial solution was combined with 5 mL of deionized water, gently stirred, and observed for bacterial activity under a microscope. Following successful staining and the optimal observation of bacterial activity, the stained bacterial suspension underwent a washing step to eliminate any residual dye, ensuring only fluorescent bacteria remained. Bacterial flagella were then sheared using a pipette through repeated aspiration until no flagella were visible under the microscope, inducing a quiescent state in the bacteria. Subsequently, the suspension was centrifuged, and an appropriate amount of MB solution was added for further use.

## 3. Results and Discussion

### 3.1. Superdiffusion of Elongated Particles in Active Flows

**Figure 2:**
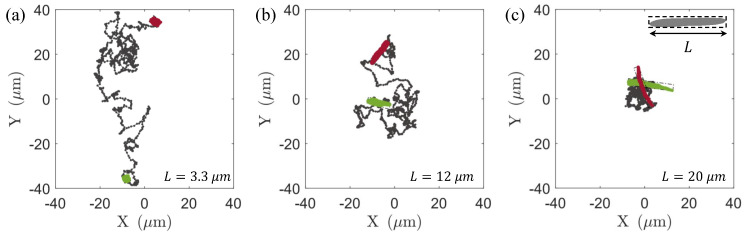
Typical trajectories of non–motile bacteria of different body lengths and the swimming bacteria with a concentration of 36 $n_{0}$, where the green bacteria shape indicates the start of the trajectory and the red bacteria shape indicates the end posture. (**a**) L=3 μm, (**b**) L=12 μm, and (**c**) L=20 μm.

To illustrate the diffusion behavior of the passive particles in active flows, the trajectories of the passive particles with varying lengths are plotted in a flow field with a concentration of $36\;n_{0}$ for a duration of 40 s, as shown in Figure 2. When the particle length is short, it exhibits a significant and strong diffusion capability, covering a considerable spatial area within the flow field. However, with an increase in the particle length, the diffusion range gradually decreases, indicating a diminishing trend in the diffusion capacity of passive particles with increasing length in the same background flow field. To quantify the results, we employed the following formulas to calculate the mean-squared displacement in translation (${\mathrm{MSD}}_{T}$) and mean-squared angular displacement (${\mathrm{MSD}}_{R}$) of the passive particles in the laboratory coordinate system.

(1) $$\begin{array}{c} {\mathrm{MSD}}_{T}=\langle r{\left(t\right)}^{2}\rangle =\langle {\left[r\left(t+t_{0}-r\left(t_{0}\right)\right)\right]}^{2}\rangle , \end{array}$$

(2) $$\begin{array}{c} {\mathrm{MSD}}_{R}=\langle \theta {\left(t\right)}^{2}\rangle =\langle {\left[\theta \left(t+t_{0}-\theta \left(t_{0}\right)\right)\right]}^{2}\rangle , \end{array}$$

**Figure 3:**
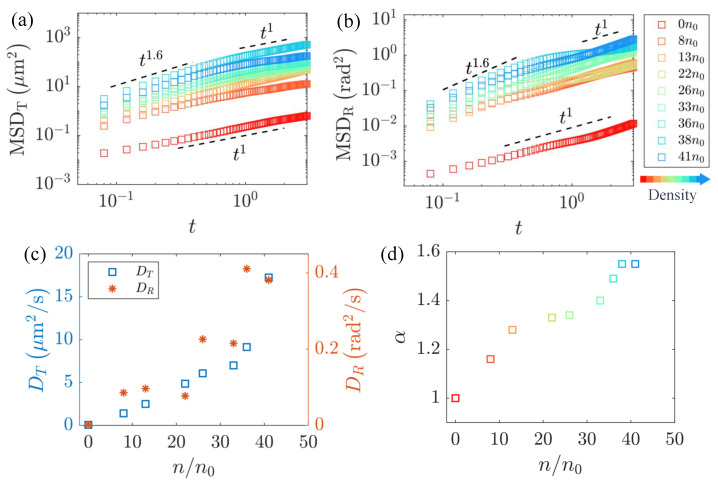
Bacterial particles with a length of 13 μm undergo translational and rotational diffusion in an active flow environment with swimming bacteria. (**a**,**b**) Translational and rotational mean-squared displacement ${\mathrm{MSD}}_{T}$, ${\mathrm{MSD}}_{R}$ of tracer particles at different bacterial concentrations. (**c**) The dependence of translational and rotational diffusion coefficient $D_{T}$, $D_{R}$ on bacterial concentration. The vertical dashed line indicates the critical density to exhibit collective motion. (**d**) The relation between superdiffusion exponent α and bacterial concentration.

Here, a rectangular bounding box encapsulates the passive particle, with the length of the box equal to the length of the passive particle and the width representing the particle’s width, as illustrated in Figure 2c; $r\left(t\right)$ and θ(t) represent the center position and orientation of the rectangular frame along its trajectory at time *t*. The two degrees of freedom exhibit qualitatively similar trends in Figure 3a,b. In the short timescale, particles undergo superdiffusive motion, characterized by mean-squared displacement (MSD) scaling as $\mathrm{MSD}∼t^{\alpha }$ with α>1. As time progresses, the motion gradually reverts to normal diffusion with α=1. The respective diffusion coefficients are defined as $D_{T}=\langle {\left[\Delta r\left(t\right)\right]}^{2}\rangle /4t$ and $D_{R}=\langle {\left[\Delta \theta \left(t\right)\right]}^{2}\rangle /2t$, as illustrated in Figure 3c. The results demonstrate that both the $D_{T}$ and $D_{R}$ values increase with the bacterial concentration, in line with the results reported in the reference literature [62,63,65].

In the flow field generated by the freely swimming bacteria, the velocity decreases with distance following a $1/r^{2}$ decay law when far from the boundaries. However, as the bacteria approach the boundaries and move near solid surfaces, the velocity decay is even faster, following a $1/r^{4}$ pattern [3,68]. If the swimmer is confined between two no-slip walls, as in our flat chamber with a height H, then the flow field becomes exponentially decayed at distances $\left|r\right|\gg H$ [69], suggesting the bacteria in the quasi-2D system with boundaries need a higher concentration to have a shorter average free path and to form collective motion under the hydrodynamic interactions between individuals [3]. Therefore, in our experiments, the relationship between the translational diffusion coefficient and the rotational diffusion coefficient with respect to the concentration did not exhibit a particularly distinct transition, as indicated in the reference literature [62]. This absence of a pronounced transition hindered our ability to determine the critical concentration at which the bacterial suspension underwent a transition from disorder to order. However, by empirical evidence, loosely speaking, about 1% of the volume fraction of the bacterial suspension was recognized as the critical concentration, above which the coherent flows were generated by the coordinated bacterial swimming [70,71]. By fitting the superdiffusive portion of the curves, we obtained the variation of the diffusion exponent α with the number density, as depicted in Figure 3d. The diffusion exponent α consistently increased monotonically with the bacterial concentration, indicating that the straight motion of the passive rod-like particles became more pronounced at higher bacterial concentrations, and the obtained results are consistent with those reported in the reference literature [33].

**Figure 4:**
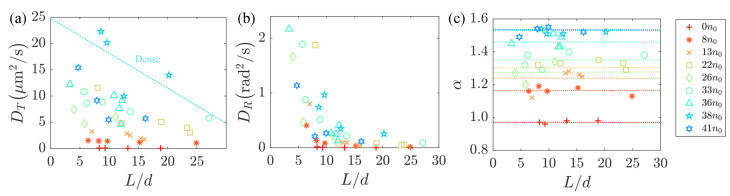
(**a**,**b**) The translational diffusion coefficient $D_{T}$ and rotational diffusion coefficient $D_{R}$ decrease with the aspect ration L/d, within active flows generated by swimming bacteria at different concentrations up to $41\;n_{0}$. (**c**) the variation of the superdiffusion exponent index α with L/d, where d=1 μm.

In order to better understand the influence of the length of the passive rod-like particles on their diffusion behavior, we introduced the antibiotic cefamandole during the cultivation of the non-motile AW405 bacteria to tune the body length. To comprehensively elucidate the influence of the passive particle length and bacterial concentration on the diffusion, we conducted calculations for the translational diffusion coefficient $D_{T}$, rotational diffusion coefficient $D_{R}$, and superdiffusion exponent α of the passive particles at different bacterial concentrations, as depicted in Figure 4. Figure 4a,b reveal a monotonic decrease in both $D_{T}$ and $D_{R}$ with an increase in particle length *L*. Since the viscous drag forces acting on moving particles are proportional to $L^{2}$ with a constant cross-section, this leads to a decrease of the diffusion coefficients with the aspect ratio. We think that the cause of this phenomenon is longer particles, and the fluid may induce greater frictional forces, which could be a primary factor contributing to the observed decrease in diffusion. Additionally, it is noteworthy that the superdiffusion exponent α of passive particles, as shown in Figure 4c, is largely associated with the background flow field and exhibits minimal dependence on particle length.

### 3.2. Anisotropic Diffusion of Elongated Particles in Active Flows

**Figure 5:**
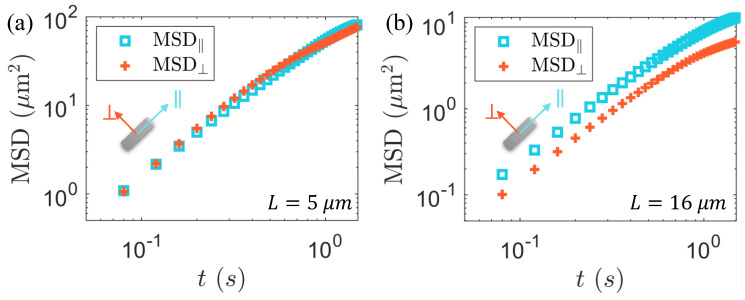
Diffusion in the moving coordinates (body frame). The MSD of two bacteria with different lengths in the body frame coordinate system at a uniform bacterial concentration of 41 $n_{0}$. (**a**) L=5 μm and (**b**) L=16 μm.

Elongated particles moving in viscous fluid experience anisotropic drag forces. An interesting question is what are the transport properties of long particles under the coupling with active coherent flow, which still remains unclear. To explore the impact of particle anisotropy on diffusion, we analyzed the mean-squared displacements (${\mathrm{MSD}}_{\|}$ and ${\mathrm{MSD}}_{⊥}$) of the passive particles along the long and short axes in the body coordinate system, as shown in Figure 5.

**Figure 6:**
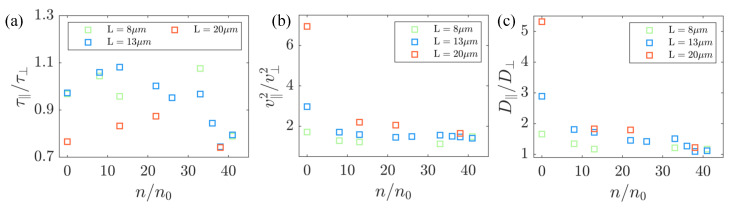
Velocity autocorrelation time projected in the parallel $\tau_{\|}$ and perpendicular $\tau_{⊥}$ direction along the major axis of the passive bacteria body in the moving body frame system. In the body coordinate system, the variations of the different lengths of the passive particles with the bacterial concentration (**a**) $v_{\|}^{2}/v_{⊥}^{2}$, (**b**) $\tau_{\|}/\tau_{⊥}$, and (**c**) $D_{\|}/D_{⊥}$ are plotted, respectively.

We constructed the total body frame displacement by summing the displacements at each small time step: $r_{i}\left(t\right)=\sum_{n=0}^{k}\Delta r_{i}\left(t_{n}\right)$, where $t_{k}=t_{0}+t$, i=‖ or ⊥. The decomposed MSD of the passive particles along the long and short axes is, respectively, denoted as ${\mathrm{MSD}}_{\|}=\langle \Delta r_{\|}\rangle$ and ${\mathrm{MSD}}_{⊥}=\langle \Delta r_{⊥}\rangle$, as illustrated in Figure 5a,b. We observed that, at the same bacterial concentration, the passive particles of short and long particles exhibited different behaviors. For a relatively short particle, a slight larger ${\mathrm{MSD}}_{⊥}$ compared to ${\mathrm{MSD}}_{\|}$ at a short timescale represents a weak preference for transporting perpendicular to its major axis. For the long particle, an apparently larger ${\mathrm{MSD}}_{\|}$ means it behaves in the opposite way. To elucidate this phenomenon, we employed the Green–Kubo formula to investigate the translational diffusion coefficients along the major and minor axes in the body-fixed coordinate system. The diffusion coefficient is defined as the integral of the velocity autocorrelation function [62,72].

(3) $$\begin{array}{c} D_{i}=\int_{0}^{\infty }\langle v_{i}\left(t_{0}+t\right)\cdot v_{i}\left(t_{0}\right)\rangle dt, \end{array}$$

where i=‖ or ⊥. Assuming a low correlation between the magnitude and direction of the velocity, Equation (3) can be expressed as follows: $D_{\|}=\langle v^{2}\rangle \int_{0}^{\infty }\langle \cos\left(\beta \left(t_{0}+t\right)\right)\cdot \cos\left(\beta \left(t_{0}\right)\right)\rangle dt$, $D_{\|}=\langle v^{2}\rangle \int_{0}^{\infty }\langle \sin\left(\beta \left(t_{0}+t\right)\right)\cdot \sin\left(\beta \left(t_{0}\right)\right)\rangle dt$, Here, β represents the angle between the particle velocity direction and its major axis and $\langle v^{2}\rangle$ denotes the kinetic energy of the particle. Furthermore, due to the exponential decay of the autocorrelation function of the velocity directions, we have: $\langle \cos\left(\beta \left(t_{0}+t\right)\right)\cdot \cos\left(\beta \left(t_{0}\right)\right)\rangle =\langle \cos^{2}\left(t_{0}\right)\rangle \cdot exp\left(-t/\tau_{\|}\right)$, $\langle \sin\left(\beta \left(t_{0}+t\right)\right)\cdot \sin\left(\beta \left(t_{0}\right)\right)\rangle =\langle \sin^{2}\left(t_{0}\right)\rangle \cdot exp\left(-t/\tau_{⊥}\right)$, where the correlation time τ represents the characteristic time of the spontaneous flow in the bacteria’s active flow. Finally, the ratio of the diffusion coefficients can be expressed as: $D_{\|}/D_{⊥}=v_{\|}^{2}/v_{⊥}^{2}\cdot \tau_{\|}/\tau_{\|}$. The combined effects of these parameters result in the observed differences in the diffusion coefficients, as illustrated in Figure 6. In Figure 6a, we observe that the ratio of the correlation time along the two axes shows a slight decay with strong fluctuations. This differs from the results in previous work [62], in which the time ratio monotonically goes from greater than 1 to less than 1 along the bacterial concentration. We believe that this difference is due to the irregularity of the shape of the passive bacteria, compared to controllable ellipsoidal particles. Additional, in Figure 6b, the parameter $v_{\|}^{2}/v_{⊥}^{2}$ is greater than 1 at low concentrations, gradually decreasing to approach a constant value around 1 at high concentrations. However, in the current work, $v_{\|}^{2}/v_{⊥}^{2}$ did not exhibit values less than 1. This observation is primarily attributed to the confinement of the chamber utilized in our experiments. This constraint restricts the correlation length of the background flow field, resulting in a scarcity of passive bacteria with body lengths much smaller than the correlation length. However, the ratio of the translational diffusion coefficients along the long and short axes decreases with increasing bacterial concentration in Figure 6c, consistent with previous work.

**Figure 7:**
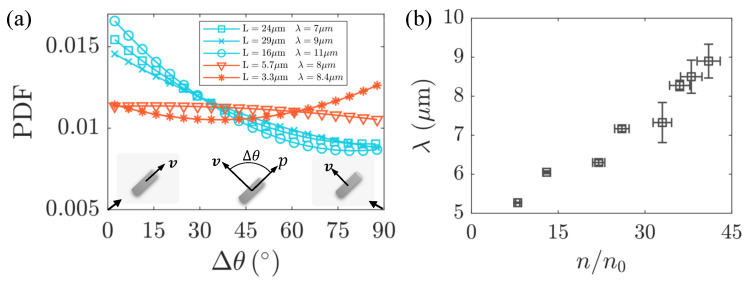
Motion of the passive particle along its long axis depending on the flow structure of the active bath. (**a**) Probability Density Function (PDF) distribution of the absolute values of the angles between the long axis of the passive particles and the velocity direction. (**b**) Variation of the correlation length λ of bacterial active flows with the bacterial concentration.

To comprehensively understand the observed phenomenon, we speculated that it is likely related to the flow structure of the background flow field. Therefore, we measured the correlation length of the background flow field at different bacterial concentrations, as shown in Figure 7b. To further validate our hypothesis, in the laboratory coordinate system, we measured the absolute angle Δθ between the long axis *p* of passive particles and the instantaneous velocity direction. The probability density distribution of Δθ is illustrated in Figure 7a. If an ellipsoidal particle moves along its major axis, $\Delta \theta \approx 0^{\circ }$. Conversely, $\Delta \theta \approx 90^{\circ }$ corresponds to the transverse motion of the passive particles. We observed that, when the length *L* of the passive particles exceeds the correlation length λ of the active flow, the probability density distribution peaks at $\Delta \theta \approx 0^{\circ }$, indicating a preference for diffusion along the long axis. Conversely, when *L* is less than λ, the peak of the probability density distribution gradually shifts towards $\Delta \theta \approx 90^{\circ }$, suggesting a tendency for diffusion along the short axis.

**Figure 8:**
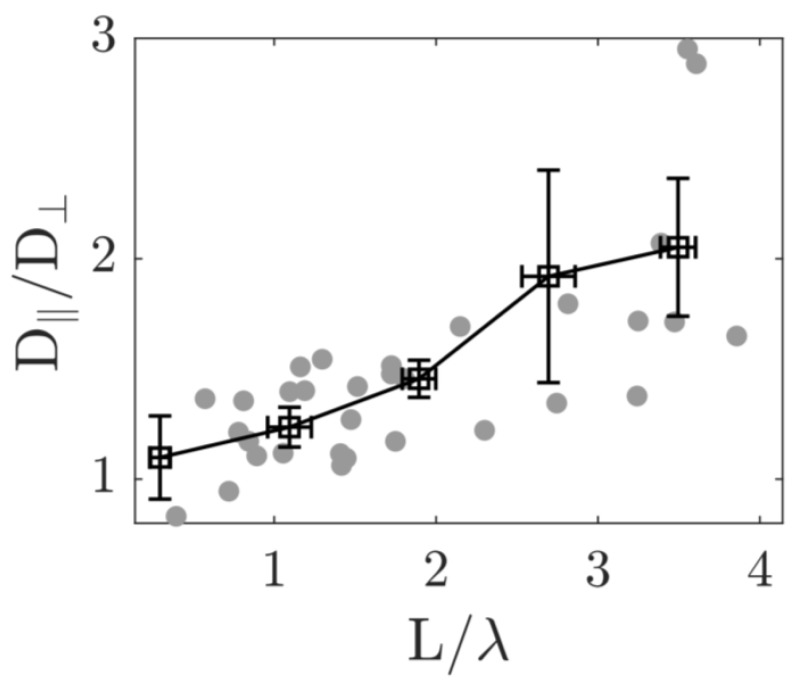
Anisotropic diffusion of passive elongated particle in active flow with the compliance between the particle geometry and active flow with different coherent lengths. In the bacterial active flow, the ratio of the anisotropic diffusion coefficients $D_{\|}/D_{⊥}$ of the passive particles in the body coordinate system varies with the ratio of the length *L* to the fluid correlation length λ.

The analysis suggests that the length of the passive particles *L* and the correlation length λ of the background flow field are crucial parameters determining the direction of passive particle diffusion. Subsequently, we related the anisotropic diffusion of the passive particles to the flow structure of the active flow and studied the ratio $D_{\|}/D_{⊥}$ as a function of L/λ. The results in Figure 8 show that $D_{\|}/D_{⊥}$ increases monotonically with L/λ. This indicates that, when L/λ is small, i.e., when the particle length is much smaller than the correlation length of the background flow field, the particle is influenced by a single coherent stream in the active flow, which drives the particle motion along the short axis. For particles with a length greater than the correlation length, they are simultaneously influenced by multiple streams, causing the passive particles to preferentially move along their long axis.

The observed phenomena suggest a complex interplay between bacterial concentration, particle length, and diffusion behavior. The superdiffusive motion of passive rod-like particles is influenced by bacterial concentration, with higher concentrations leading to more-pronounced superdiffusion. Additionally, the particle length plays a crucial role in determining the translational and rotational diffusion coefficients, while maintaining the robustness of superdiffusion. The anisotropic diffusion observed at different bacterial concentrations and particle lengths highlights the intricate coupling between particle motion and the surrounding active flow. The dependence of the anisotropic diffusion on the correlation length of the active flow provides valuable insights into the underlying physics governing the behavior of passive rod-like particles in bacterial baths.

The current theoretical predictions and experimental validations indicate that the aspect ratio of self-propelled particles plays a crucial role in their collective behavior [73]. Despite their relatively low abundance, longer bacteria contribute to the aggregation process at lower densities by aligning themselves, enhancing cooperative motion and facilitating large-scale global superdiffusion. However, due to the substantial number of bacteria involved in the collective motion, studying an individual bacterium within the cluster poses challenges. Bacterial motion is not only influenced by neighboring bacteria, but also by their own self-propulsion. In examining a single bacterium within a cluster, challenges arise as its velocity is influenced by its intrinsic characteristics, while the surrounding bacteria also exert an influence.

## 4. Conclusions

We have presented an experimental study on the transport of passive anisotropic particles in active flow generated by motile bacteria. We found, consistent with spherical particles, a superdiffusion at a short time and returning back to normal diffusion at a long time. The translational and rotational diffusion coefficients decay as the length increases. Besides this, the transport of these passive particles is not only influenced by their length *L*, but also related to the correlation length of the background active flow λ.

Specifically, when the length of the passive particles was greater than the correlation length of the active flow, we found that the particles exhibited faster diffusion along their main axis, resembling the dynamics of ellipsoids undergoing Brownian motion. Conversely, when the length of passive particles was smaller than the correlation length of the flow filed, the particles showed faster diffusion along the transverse direction of their main axis. Furthermore, we investigated the coupling between the anisotropic diffusion of the passive particles and the flow structure of the active flow. By studying the ratio of the transport diffusion coefficient along the main axis $D_{\|}$ to the transverse diffusion coefficient $D_{⊥}$ as a function of the length-to-fluid correlation-length ratio L/λ, we found that both $D_{\|}/D_{⊥}$ monotonically increased with the increase in L/λ. Our investigation addressed the gap in understanding anisotropic particle dynamics in active coherent flows, providing novel insights into the interplay between particle morphology, flow structure, concentration, and diffusion behavior.

## Data Availability

Data are contained within the article.

## References

[1] Zhang H.P., Be’er A., Florin E.L., Swinney H.L. (2010). Collective motion and density fluctuations in bacterial colonies. Proc. Natl. Acad. Sci. USA.

[2] Leptos K.C., Guasto J.S., Gollub J.P., Pesci A.I., Goldstein R.E. (2009). Dynamics of enhanced tracer diffusion in suspensions of swimming eukaryotic microorganisms. Phys. Rev. Lett..

[3] Wensink H.H., Dunkel J., Heidenreich S., Drescher K., Goldstein R.E., Löwen H., Yeomans J.M. (2012). Meso-scale turbulence in living fluids. Proc. Natl. Acad. Sci. USA.

[4] Saintillan D., Shelley M.J. (2007). Orientational order and instabilities in suspensions of self-locomoting rods. Phys. Rev. Lett..

[5] Bratanov V., Jenko F., Frey E. (2015). New class of turbulence in active fluids. Proc. Natl. Acad. Sci. USA.

[6] Duclos G., Erlenkämper C., Joanny J.F., Silberzan P. (2017). Topological defects in confined populations of spindle-shaped cells. Nat. Phys..

[7] Wu K.T., Hishamunda J.B., Chen D.T., DeCamp S.J., Chang Y.W., Fernández-Nieves A., Fraden S., Dogic Z. (2017). Transition from turbulent to coherent flows in confined three-dimensional active fluids. Science.

[8] Pérez Estay B.I. (2022). Characterization of *E. coli* Swimming Near Sinusoidal Surfaces. Master’s Thesis.

[9] Toner J., Tu Y. (1998). Flocks, herds, and schools: A quantitative theory of flocking. Phys. Rev. E.

[10] Ballerini M., Cabibbo N., Candelier R., Cavagna A., Cisbani E., Giardina I., Lecomte V., Orlandi A., Parisi G., Procaccini A. (2008). Interaction ruling animal collective behavior depends on topological rather than metric distance: Evidence from a field study. Proc. Natl. Acad. Sci. USA.

[11] Toner J., Guttenberg N., Tu Y. (2018). Swarming in the dirt: Ordered flocks with quenched disorder. Phys. Rev. Lett..

[12] Couzin I.D., Franks N.R. (2003). Self-organized lane formation and optimized traffic flow in army ants. Proc. R. Soc. Lond. Ser. Biol. Sci..

[13] Bazazi S., Buhl J., Hale J.J., Anstey M.L., Sword G.A., Simpson S.J., Couzin I.D. (2008). Collective motion and cannibalism in locust migratory bands. Curr. Biol..

[14] Becco C., Vandewalle N., Delcourt J., Poncin P. (2006). Experimental evidences of a structural and dynamical transition in fish school. Phys. Stat. Mech. Appl..

[15] Makris N.C., Ratilal P., Jagannathan S., Gong Z., Andrews M., Bertsatos I., Godø O.R., Nero R.W., Jech J.M. (2009). Critical population density triggers rapid formation of vast oceanic fish shoals. Science.

[16] Bricard A., Caussin J.B., Desreumaux N., Dauchot O., Bartolo D. (2013). Emergence of macroscopic directed motion in populations of motile colloids. Nature.

[17] Vicsek T., Czirók A., Ben-Jacob E., Cohen I., Shochet O. (1995). Novel type of phase transition in a system of self-driven particles. Phys. Rev. Lett..

[18] Grégoire G., Chaté H. (2004). Onset of collective and cohesive motion. Phys. Rev. Lett..

[19] Narayan V., Ramaswamy S., Menon N. (2007). Long-lived giant number fluctuations in a swarming granular nematic. Science.

[20] Nagai K.H., Sumino Y., Montagne R., Aranson I.S., Chaté H. (2015). Collective motion of self-propelled particles with memory. Phys. Rev. Lett..

[21] Di Leonardo R. (2016). Controlled collective motions. Nat. Mater..

[22] Sanchez T., Chen D.T., DeCamp S.J., Heymann M., Dogic Z. (2012). Spontaneous motion in hierarchically assembled active matter. Nature.

[23] Schaller V., Bausch A.R. (2013). Topological defects and density fluctuations in collectively moving systems. Proc. Natl. Acad. Sci. USA.

[24] Einstein A. (1905). On the motion of small particles suspended in liquids at rest required by the molecular-kinetic theory of heat. Ann. Phys..

[25] Libchaber A. (2019). From biology to physics and back: The problem of Brownian movement. Annu. Rev. Condens. Matter Phys..

[26] Sokolov A., Goldstein R.E., Feldchtein F.I., Aranson I.S. (2009). Enhanced mixing and spatial instability in concentrated bacterial suspensions. Phys. Rev. E.

[27] Kurtuldu H., Guasto J.S., Johnson K.A., Gollub J.P. (2011). Enhancement of biomixing by swimming algal cells in two-dimensional films. Proc. Natl. Acad. Sci. USA.

[28] Bechinger C., Di Leonardo R., Löwen H., Reichhardt C., Volpe G., Volpe G. (2016). Active particles in complex and crowded environments. Rev. Mod. Phys..

[29] Saintillan D. (2018). Rheology of active fluids. Annu. Rev. Fluid Mech..

[30] Zhu Z., Liu Q.X. (2019). Enhanced transport of nutrients powered by microscale flows of the self-spinning dinoflagellate *Symbiodinium* sp.. J. Exp. Biol..

[31] Ye S., Liu P., Ye F., Chen K., Yang M. (2020). Active noise experienced by a passive particle trapped in an active bath. Soft Matter.

[32] Granek O., Kafri Y., Tailleur J. (2022). Anomalous transport of tracers in active baths. Phys. Rev. Lett..

[33] Xie C., Liu Y., Luo H., Jing G. (2022). Activity-Induced Enhancement of Superdiffusive Transport in Bacterial Turbulence. Micromachines.

[34] Ning L., Lou X., Ma Q., Yang Y., Luo N., Chen K., Meng F., Zhou X., Yang M., Peng Y. (2023). Hydrodynamics-Induced Long-Range Attraction between Plates in Bacterial Suspensions. Phys. Rev. Lett..

[35] Ariel G., Rabani A., Benisty S., Partridge J.D., Harshey R.M., Be’Er A. (2015). Swarming bacteria migrate by Lévy Walk. Nat. Commun..

[36] Wen X., Sang Y., Zhang Y., Ge F., Jing G., He Y. (2023). Direct Observation of Nanotracer Transport in Swarming Bacteria during Antibiotic Adaptation. ACS Nano.

[37] Wu X.L., Libchaber A. (2000). Particle diffusion in a quasi-two-dimensional bacterial bath. Phys. Rev. Lett..

[38] Kim M.J., Breuer K.S. (2004). Enhanced diffusion due to motile bacteria. Phys. Fluids.

[39] Chen D.T., Lau A., Hough L.A., Islam M.F., Goulian M., Lubensky T.C., Yodh A.G. (2007). Fluctuations and rheology in active bacterial suspensions. Phys. Rev. Lett..

[40] Mino G., Mallouk T.E., Darnige T., Hoyos M., Dauchet J., Dunstan J., Soto R., Wang Y., Rousselet A., Clement E. (2011). Enhanced diffusion due to active swimmers at a solid surface. Phys. Rev. Lett..

[41] Wilson L.G., Martinez V.A., Schwarz-Linek J., Tailleur J., Bryant G., Pusey P., Poon W.C. (2011). Differential dynamic microscopy of bacterial motility. Phys. Rev. Lett..

[42] Miño G., Dunstan J., Rousselet A., Clément E., Soto R. (2013). Induced diffusion of tracers in a bacterial suspension: Theory and experiments. J. Fluid Mech..

[43] Jepson A., Martinez V.A., Schwarz-Linek J., Morozov A., Poon W.C. (2013). Enhanced diffusion of nonswimmers in a three-dimensional bath of motile bacteria. Phys. Rev. E.

[44] Patteson A.E., Gopinath A., Purohit P.K., Arratia P.E. (2016). Particle diffusion in active fluids is non-monotonic in size. Soft Matter.

[45] Valeriani C., Li M., Novosel J., Arlt J., Marenduzzo D. (2011). Colloids in a bacterial bath: Simulations and experiments. Soft Matter.

[46] Vaccari L., Allan D.B., Sharifi-Mood N., Singh A.R., Leheny R.L., Stebe K.J. (2015). Films of bacteria at interfaces: Three stages of behaviour. Soft Matter.

[47] Gupta V., Singla R., Singh G., Chanda A. (2023). Development of Soft Composite Based Anisotropic Synthetic Skin for Biomechanical Testing. Fibers.

[48] Vakhrusheva A., Murashko A., Trifonova E., Efremov Y.M., Timashev P., Sokolova O. (2022). Role of actin-binding proteins in the regulation of cellular mechanics. Eur. J. Cell Biol..

[49] Duggal R., Pasquali M. (2006). Dynamics of individual single-walled carbon nanotubes in water by real-time visualization. Phys. Rev. Lett..

[50] Cheong F.C., Grier D.G. (2010). Rotational and translational diffusion of copper oxide nanorods measured with holographic video microscopy. Opt. Express.

[51] Bhaduri B., Neild A., Ng T.W. (2008). Directional Brownian diffusion dynamics with variable magnitudes. Appl. Phys. Lett..

[52] Kraft D.J., Wittkowski R., Ten Hagen B., Edmond K.V., Pine D.J., Löwen H. (2013). Brownian motion and the hydrodynamic friction tensor for colloidal particles of complex shape. Phys. Rev. E.

[53] Maragó O.M., Bonaccorso F., Saija R., Privitera G., Gucciardi P.G., Iatì M.A., Calogero G., Jones P.H., Borghese F., Denti P. (2010). Brownian motion of graphene. ACS Nano.

[54] Köster S., Steinhauser D., Pfohl T. (2005). Brownian motion of actin filaments in confining microchannels. J. Phys. Condens. Matter.

[55] Yang J., Francois N., Punzmann H., Shats M., Xia H. (2019). Diffusion of ellipsoids in laboratory two-dimensional turbulent flow. Phys. Fluids.

[56] Perrin F. (1934). Mouvement brownien d’un ellipsoide-I. Dispersion diélectrique pour des molécules ellipsoidales. J. Phys. Radium.

[57] Perrin F. (1936). Mouvement Brownien d’un ellipsoide (II). Rotation libre et dépolarisation des fluorescences. Translation et diffusion de molécules ellipsoidales. J. Phys. Radium.

[58] Vasanthi R., Ravichandran S., Bagchi B. (2001). Needlelike motion of prolate ellipsoids in the sea of spheres. J. Chem. Phys..

[59] Vasanthi R., Bhattacharyya S., Bagchi B. (2002). Anisotropic diffusion of spheroids in liquids: Slow orientational relaxation of the oblates. J. Chem. Phys..

[60] Han Y., Alsayed A.M., Nobili M., Zhang J., Lubensky T.C., Yodh A.G. (2006). Brownian motion of an ellipsoid. Science.

[61] Han Y., Alsayed A., Nobili M., Yodh A.G. (2009). Quasi-two-dimensional diffusion of single ellipsoids: Aspect ratio and confinement effects. Phys. Rev. E.

[62] Peng Y., Lai L., Tai Y.S., Zhang K., Xu X., Cheng X. (2016). Diffusion of ellipsoids in bacterial suspensions. Phys. Rev. Lett..

[63] Yang O., Peng Y., Liu Z., Tang C., Xu X., Cheng X. (2016). Dynamics of ellipsoidal tracers in swimming algal suspensions. Phys. Rev. E.

[64] Xu R.k., Jiang H.j., Hou Z.h. (2021). Simulation study of passive rod diffusion in active bath: Nonmonotonic length dependence and abnormal translation-rotation coupling. Chin. J. Chem. Phys..

[65] Nordanger H., Morozov A., Stenhammar J. (2022). Anisotropic diffusion of ellipsoidal tracers in microswimmer suspensions. Phys. Rev. Fluids.

[66] Aporvari M.S., Utkur M., Saritas E.U., Volpe G., Stenhammar J. (2020). Anisotropic dynamics of a self-assembled colloidal chain in an active bath. Soft Matter.

[67] Zhang C., Xie C., Feng W., Luo H., Liu Y., Jing G. (2023). Configurational dynamics of flexible filaments in bacterial active baths. New J. Phys..

[68] Drescher K., Dunkel J., Cisneros L.H., Ganguly S., Goldstein R.E. (2011). Fluid dynamics and noise in bacterial cell–cell and cell–surface scattering. Proc. Natl. Acad. Sci. USA.

[69] Liron N., Mochon S. (1976). Stokes flow for a stokeslet between two parallel flat plates. J. Eng. Math..

[70] Gachelin J., Rousselet A., Lindner A., Clement E. (2014). Collective motion in an active suspension of Escherichia coli bacteria. New J. Phys..

[71] Yan N., Xie C., Luo H., Liu Y., Jing G. (2023). Bacterial turbulence in gradient confinement. Chin. Phys. B.

[72] Zwanzig R. (2001). Nonequilibrium Statistical Mechanics.

[73] Ilkanaiv B., Kearns D.B., Ariel G., Be’er A. (2017). Effect of cell aspect ratio on swarming bacteria. Phys. Rev. Lett..

